# Agmatine Mitigates Diabetes-Related Memory Loss in Female Mice by Targeting I_2_/I_3_ Imidazoline Receptors and Enhancing Brain Antioxidant Defenses

**DOI:** 10.3390/antiox14070837

**Published:** 2025-07-08

**Authors:** Luis E. Cobos-Puc, Hilda Aguayo-Morales

**Affiliations:** Facultad de Ciencias Químicas, Universidad Autónoma de Coahuila, Boulevard Venustiano Carranza S/N Esq. Con Ing. José Cárdenas Valdés, República Oriente, Saltillo 25280, Mexico

**Keywords:** agmatine, diabetes, female mice, memory, imidazoline receptor, antioxidants

## Abstract

Cognitive decline is a common complication of diabetes mellitus, driven in part by oxidative stress and impaired glucose–insulin homeostasis. This study examined the neuroprotective effects of agmatine (200 mg/kg intraperitoneally) in female BALB/c diabetic mice. Several receptor pathways were examined using commercially available antagonists. Behavioral performance was evaluated using the novel object recognition test. Metabolic parameters, such as glucose and insulin levels, as well as antioxidants, including catalase (CAT), superoxide dismutase (SOD), and glutathione (GSH), were measured in blood and brain tissue. The diabetic mice exhibited impaired recognition memory (discrimination index = 0.08), hyperglycemia (24.3 mmol/L), decreased insulin levels (38.4 µU/mL), and diminished antioxidant defenses (CAT: 75.4 U/g tissue, SOD: 32.6 U/g tissue, and GSH: 8.3 mmol/g tissue). Agmatine treatment improved cognitive function and reversed the biochemical alterations. However, these effects were reduced when agmatine was co-administered with imidazoline I_2_/I_3_ receptor antagonists. Correlation analysis revealed that cognitive performance positively correlated with antioxidant enzyme levels and insulin levels and negatively correlated with glucose concentrations. Strong intercorrelations among CAT, SOD, and GSH levels suggest a coordinated antioxidant response. Overall, these results imply that agmatine’s neuroprotective effects are partially mediated by modulation of the oxidative balance and glucose–insulin regulation via imidazoline receptors.

## 1. Introduction

Diabetes mellitus is a chronic metabolic disease characterized by sustained hyperglycemia. It affects millions of people worldwide. Classic complications include peripheral neuropathy, nephropathy, and cardiovascular disease. However, there is growing evidence linking diabetes to progressive cognitive decline and an increased risk of developing neurodegenerative diseases, such as Alzheimer’s disease (AD) [1,2]. Chronic hyperglycemia creates a pro-oxidative environment in the brain. This environment is characterized by the excessive production of reactive oxygen species (ROS), altered synaptic plasticity, and disrupted brain insulin signaling [3,4]. The vulnerability caused by chronic hyperglycemia is exacerbated by the limited antioxidant capacity of the central nervous system (CNS) [5]. Therefore, strengthening endogenous defenses, such as catalase (CAT), superoxide dismutase (SOD), and reduced glutathione (GSH), is crucial for preserving cognitive function.

Agmatine is a biogenic amine synthesized from L-arginine by the enzyme arginine decarboxylase. This metabolic pathway is present in bacteria, mammals, and neuronal tissues. It is primarily produced in the intestine, liver, and CNS. High concentrations of agmatine are found in alcoholic beverages, such as beer, wine, and sake, as well as in fermented foods. Tissue agmatine levels are regulated by its conversion to either putrescine via agmatinase or γ-guanidinobutyraldehyde via diamine oxidase [6]. Agmatine has notable effects on the brain. For example, it enhances the analgesic effects of opioids and exhibits direct antinociceptive properties [7]. Agmatine has also been studied as an adjunctive treatment for mood disorders, such as depression, due to its ability to enhance the action of antidepressants by modulating the monoaminergic axis and promoting hippocampal neurogenesis [8]. The cerebral actions of agmatine are mediated by a complex network of effectors including N-methyl-D-aspartate (NMDA) receptors, opioid receptors, serotonin receptors, α_2_-adrenoceptors, imidazoline receptors, and nitric oxide [6,7,8]. In the cardiovascular system, agmatine activates imidazoline I_1_ receptors in the medulla oblongata, thereby reducing sympathetic activity and blood pressure [9]. Furthermore, agmatine has been reported to inhibit macrophage activation and proinflammatory cytokine production [10,11], positioning it as a potential modulator of autoimmune and chronic inflammatory diseases.

It has been extensively studied for its neuroprotective, antioxidant, and anti-inflammatory properties [12,13]. Several studies have demonstrated agmatine’s ability to improve memory, reduce neuroinflammation, and enhance synaptic plasticity, particularly in male, non-diabetic models [14,15,16]. However, these studies have not been conducted on female populations or complex pathophysiological conditions, such as diabetes. Therefore, it is crucial to analyze whether the beneficial effects of agmatine apply to organisms that are more susceptible to cognitive decline, such as diabetic females. Women with diabetes, for instance, are at a significantly higher risk of cognitive impairment than men. This is partly due to hormonal differences, particularly the decline in estrogen during menopause. This decline leads to increased oxidative stress, inflammation, and synaptic dysfunction [17,18,19]. In animal models, memory deficits are accentuated during phases of the estrous cycle with low estrogen levels, such as metestrus and diestrus [20]. Thus, the present study employed an experimental model of female BALB/c mice with streptozotocin (STZ)-induced type 1 diabetes. The mice were evaluated during the metestrus and diestrus phases of the estrous cycle. This design allowed us to model a critical scenario in which three risk factors converge: hyperglycemia, estrogen deficiency, and cerebral oxidative stress.

One might think that using a type 1 diabetes model would limit the applicability of the findings since most people have type 2 diabetes. However, this model was chosen because it quickly and consistently induces diabetes. Furthermore, this model does not involve obesity, peripheral insulin resistance, or dyslipidemia, all of which are all common in type 2 diabetes [21]. This allows the direct effects of hyperglycemia on memory and brain oxidative stress to be isolated. The novel object recognition test (NORT), on the other hand, is a widely used behavioral model for assessing recognition memory in rodents by capitalizing on their innate curiosity about new objects. The NORT primarily measures short- and long-term memory, allowing researchers to study cognitive deficits associated with neurodegenerative diseases, neurological disorders, and the effects of drugs or therapeutic interventions. It is a non-invasive, highly reproducible test that evaluates the function of key regions involved in non-spatial memory function [22,23,24]. Based on this, the focus of this study is to evaluate the effects of agmatine on the recognition memory of diabetic female mice using the NORT. To investigate this, we selected female mice with low estrogen levels (in the metestrus or diestrus phases) and examined whether intraperitoneal administration of agmatine at two different times would restore the discrimination index of diabetic mice. After treating the animals with agmatine alone or with antagonists of α_2_-adrenoceptors and I_1_, I_2_, and I_3_ imidazoline receptors, we measured their brain levels of SOD, CAT, GSH, and glucose, as well as their blood glucose and insulin levels.

## 2. Materials and Methods

### 2.1. Chemical Reagents and Materials

Agmatine sulfate salt, rauwolscine hydrochloride, sodium citrate dihydrate, citric acid, an insulin (RAB0817) ELISA kit, a glucose kit (MAK476), and a catalase (CAT, MAK531) colorimetric kit were purchased from Sigma Chemical Co., St. Louis, MO, USA. AGN 192403, idazoxan, and KU14R were purchased from Tocris Bioscience, Minneapolis, MN, USA. Streptozotocin (STZ) was purchased from Cayman Chemical, Ann Arbor, MI, USA. A glutathione (GSH, K006-H1) colorimetric kit was purchased from Arbor Assays, Ann Arbor, MI, USA. A Giemsa stain kit (64843) was purchased from Hycel, Zapopan, JAL, Mexico. Physiological saline solution (NaCl, 0.09%) and isoflurane were purchased from PISA Pharmaceuticals, Guadalajara, JAL, Mexico. An Accu-Chek glucometer and glucose test strips were purchased from Roche, Miguel Hidalgo, CDMX, Mexico. For the bases (agmatine and rauwolscine), the listed doses refer to the free base form. All compounds were prepared on the day of use. They were dissolved in physiological saline solution except for KU14R, which was dissolved in 10% dimethyl sulfoxide (DMSO), and STZ, which was dissolved in a citrate buffer solution.

### 2.2. Animals

A total of 132 six-month-old female BALB/c mice weighing between 27 and 33 g were purchased from the Morelos vivarium (Cuernavaca, MOR, Mexico). The mice were allowed to acclimate for one week before the start of this study. Then, the animals were randomly housed in groups of three in acrylic rodent cages containing wood shavings and no additional enrichment. The light/dark cycle was reversed at 20:00 h, and the temperature was maintained at 23 ± 2 °C with 50–70% relative humidity. The mice had ad libitum access to tap water and SAFE^®^ A30 food. To induce type 1 diabetes with STZ and measure blood glucose levels, the animals were fasted for 4 h beforehand. The mice were also fasted overnight before sacrifice to prevent interference with the biochemical measurements. However, they always had access to water. For behavioral, locomotor activity, or estrous cycle studies, the animals were not fasted. To minimize fear and discomfort, all handling procedures were performed by trained personnel. Injections and sampling were carried out gently and consistently to prevent tissue damage and pain. All research protocols were approved by the Ethics Committee of our institution (protocol TMCB-22-09-23-2 approved on 22 September 2023), and this study adhered to the ARRIVE guidelines for the care and use of animals in research (see Table S1) [25].

### 2.3. Estrous Cycle Determination

**Figure 1:**
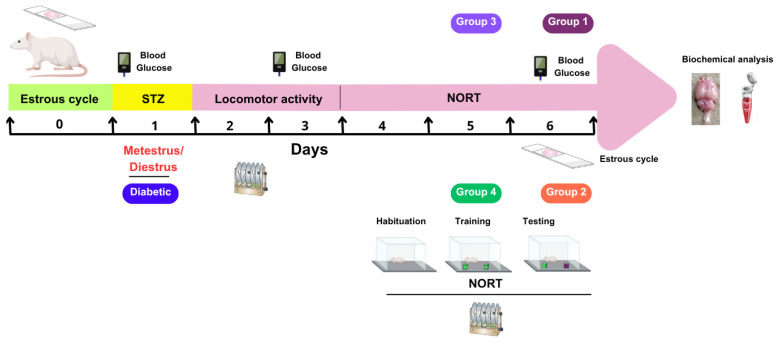
Schematic representation of the experimental protocol. STZ, streptozotocin; NORT, novel object recognition test; Agm, agmatine.

Before assigning the animals (*n* = 132) to experimental groups, their estrous cycles were determined. This was performed by inserting a pipette containing 100 µL of sterile saline solution (NaCl, 0.9%) into the vaginal orifice. The cells were then rinsed at least three times. The samples were then spread on a slide and air-dried. Finally, the samples were stained with Giemsa and examined under the microscope to determine the phase of the estrous cycle based on the predominant cell morphology [26]. This procedure was repeated for 14 consecutive days at 9:00 h. The animals were grouped on day 0 so that each experimental evaluation would coincide with the metestrus/diestrus phase on day 1, when estradiol concentrations are lower. On day 6, a vaginal smear was performed on each animal to confirm its stage of the estrous cycle (see Figure 1).

### 2.4. Diabetes Induction

On day 1, the animals (*n* = 132) received a single intraperitoneal injection of streptozotocin (STZ) at a dose of 175 mg/kg [21]. The STZ was dissolved in a citrate buffer solution containing sodium citrate dihydrate and citric acid at a concentration of 0.1 M and a pH of 4.5. Mice were considered diabetic if their fasting glucose level was 11.1 mmol/L or higher by day 3 measured with a glucometer.

### 2.5. Determination of Motor Coordination

We evaluated locomotor coordination using a rotarod apparatus (Yamto Instruments, Hyderabab, TG, India). First, the animals (*n* = 132) underwent training on the rod at 4 rpm for 60 s on day 1. On days 2–6, the speed of the rod increased from 4 to 40 rpm over 300 s, and the latency to fall off the rotarod was recorded [27]. On days 3–6, the animals rested for 30 min at the end of the corresponding phase of the NORT.

### 2.6. Novel Object Recognition Test (NORT)

The NORT was performed in a 40 × 40 × 40 cm Plexiglas box over the course of three consecutive days. The test consisted of three phases: habituation (day 4; 5 min), training (day 5; 10 min), and testing (day 6; 10 min). All trials took place in a dark procedure room lit by a red light. On the first day, each mouse was first allowed to acclimate to the arena without any objects present. The next day, two identical objects were placed in the arena during the training phase. During the testing phase, one of the objects was replaced with a novel object that differed in material and shape. The box and objects were cleaned with 70% ethanol and dried between each mouse to avoid olfactory cues. The exploratory behavior of each mouse was recorded using an infrared video camera. Object exploration was defined as sniffing or touching an object with the nose or paws or pointing the nose at an object from a distance of less than one centimeter. Sitting on or around objects was not considered exploratory behavior. An observer blinded to the treatments analyzed the recordings and manually recorded how long each mouse spent exploring the objects at each stage. The discrimination index was calculated from the recorded exploration time using the following equation [28]:

$$Discrimination\;index=\frac{T2-T1}{T2+T1}$$

where T1 is the time spent exploring the known object and T2 is the time spent exploring the new object, both in seconds.

A total of 132 animals were randomly divided into four experimental groups. Group 1 (*n* = 12) was subdivided into two subgroups that received either STZ (175 mg/kg) or citrate buffer. Each of these subgroups and the other subgroups contained six animals. Groups 2 and 3 (*n* = 42 each) were further divided into seven subgroups. These subgroups received different treatments: (1) non-diabetic animals treated with citrate buffer, (2) untreated diabetic group (DG), (3) DG treated with saline, (4) DG treated with agmatine at 50 mg/kg, (5) DG treated with agmatine at 100 mg/kg, (6) DG treated with agmatine at 200 mg/kg, and (7) DG treated with agmatine at 400 mg/kg. The difference between Groups 2 and 3 was the timing of treatment administration: animals in Group 2 received their respective treatments 15 min before the test phase of the NORT, while those in Group 3 received the treatments 24 h before the NORT. Group 4 (*n* = 36) was used to evaluate whether the memory-enhancing effect of agmatine (200 mg/kg, administered 24 h before the NORT) was influenced by different receptor antagonists at doses high enough to block their respective receptors. This group was divided into six subgroups. Each subgroup received an injection of one of the following substances 15 min before receiving agmatine administration: (1) saline as a vehicle control for the most of antagonists, (2) dimethyl sulfoxide (DMSO, 10%) as a vehicle control for KU14R, (3) rauwolscine (α_2_; 0.3 mg/kg) [29], (4) AGN 192403 (I_1_; 3 mg/kg) [29], (5) idazoxan (I_2_; 3 mg/kg) [30], and (6) KU14R (I_3_; 8 mg/kg) [31]. All referred doses were administered intraperitoneally at 100 µL.

### 2.7. Biochemical Determinations

Once the evaluations were complete, the mice were euthanized while under deep anesthesia with 4% isoflurane. The adequacy of the anesthesia was confirmed by the absence of pedal and corneal reflexes before intracardiac exsanguination. This method ensured a humane and irreversible death in accordance with ethical standards. Blood was collected in tubes containing EDTA for plasma separation and insulin quantification. Next, an incision was made in the thoracic cavity, and a catheter was inserted into the left ventricle. Then, the right atrium was cut, and phosphate-buffered saline (PBS) was perfused until all the remaining blood was completely removed. The entire brain (0.45 ± 0.23 g) was dissected from each mouse, frozen at −80 °C, and stored in PBS until use. The tissue was cut into smaller pieces using a manual homogenizer and transferred to 50 mL conical tubes containing a lysis buffer solution (50 mM Tris-HCl at pH 7.4, 150 mM NaCl, 1% Triton X-100, and a protease inhibitor cocktail) at a mass-to-volume ratio of 1:10. The tubes were shaken on ice at 300 rpm for 30 min. The resulting mixture was then centrifuged at 15,000× *g* for 20 min at 4 °C [32]. Glucose, insulin, superoxide dismutase (SOD), catalase (CAT), and glutathione (GSH) were quantified according to the manufacturer’s instructions. Since agmatine exhibited neuroprotective effects when administered 24 h before behavioral testing, a total of 78 mice were selected for biochemical analysis. The experimental groups were as follows: non-diabetic controls (Group 1); untreated diabetic controls (Group 2); and diabetic mice treated with either saline (Group 3) or agmatine at four different doses (Groups 4–7). The analysis also included diabetic mice that were pretreated with a vehicle (Groups 8–9) or with one of four different receptor antagonists (Groups 10–13), all of which received agmatine at 200 mg/kg. Each group consisted of 6 animals.

### 2.8. Statistical Analysis

All data are expressed as the mean ± standard error of the mean (SEM). All datasets met the assumption of normal distribution, as assessed by the Shapiro–Wilk test. To analyze the effects of STZ on blood glucose and locomotor activity over time, we performed a repeated-measures ANOVA followed by a Tukey post hoc test. For the discrimination index and biochemical analyses, we performed a one-way ANOVA followed by a Tukey post hoc test. We constructed a correlation matrix to examine the linear relationships among key behavioral, metabolic, and antioxidant variables measured in our experimental model. Specifically, we analyzed the associations between blood and brain glucose levels, serum and brain insulin levels, and the following antioxidants: catalase, superoxide dismutase, glutathione, and the discrimination index from the NORT. The dataset included individual values for each mouse across all experimental groups: non-diabetic controls, diabetic controls, and agmatine-treated diabetic mice (200 mg/kg). We then calculated Pearson’s correlation coefficient (r). A *p*-value of less than 0.05 was considered statistically significant in all cases. All statistical analyses were performed using GraphPad Prism version 8.0 (GraphPad Software, San Diego, CA, USA).

## 3. Results

### 3.1. Determination of the Estrous Phase in Female Mice

**Figure 2:**
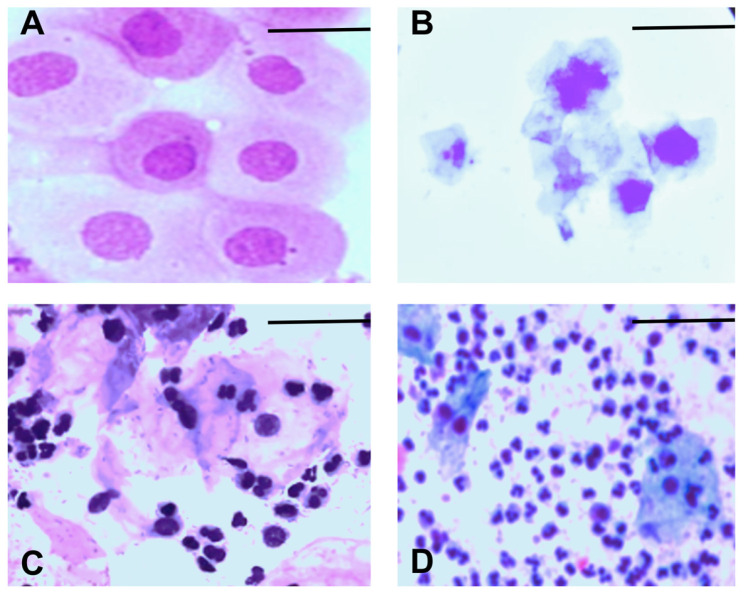
Representative images of the estrous cycle in female mice: (**A**) proestrus, (**B**) estrus, (**C**) metestrus, (**D**) diestrus. Scale bar = 10 µm. The magnification is 40×.

We monitored the estrous cycle of each animal for 14 days before grouping them into their respective experimental groups. We used the following criteria to determine the stage of the estrous cycle: proestrus, which is characterized by mostly nucleated epithelial cells (Figure 2A); estrus, which is characterized by mostly cornified epithelial cells (Figure 2B); metestrus, which is characterized by cornified epithelial cells and leukocytes (Figure 2C); and diestrus, which is characterized by mostly leukocytes (Figure 2D).

These criteria allowed us to select animals in the metestrus or diestrus phase. This ensured that the animals would be in the diestrus phase on day six, when we performed the NORT (see Table 1). Both phases are characterized by decreased estradiol levels, which are associated with impaired cognitive processes, including memory. These criteria also allowed us to evaluate the effect of diabetes on the memory of female mice under these conditions.

### 3.2. Effect of Streptozotocin on Memory in Female Mice

**Figure 3:**
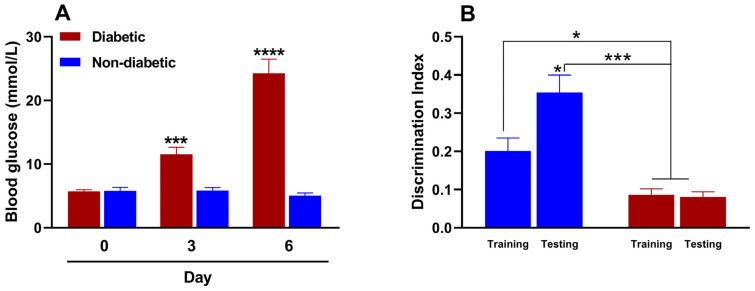
(**A**) Effect of streptozotocin (for diabetic mice; 175 mg/kg, intraperitoneally [i.p.]) or citrate buffer (for non-diabetic mice; 100 µL, i.p.) on blood glucose and (**B**) memory in female mice (*n* = 6 for each group). *, *p* < 0.05; ***, *p* < 0.001; ****, *p* < 0.001 versus the control group in metabolic and cognitive assays.

STZ-induced diabetes develops rapidly within two to three days, and many protocols primarily involve a single high dose or repeated low doses. We preliminarily evaluated the efficacy of various doses of STZ (150–200 mg/kg) when administered intraperitoneally to induce type 1 diabetes in female mice. The 150 mg/kg dose induced diabetes in only 33.3% of the animals, whereas the 200 mg/kg dose induced diabetes in all of the treated animals. However, the 200 mg/kg dose resulted in 100% lethality rate by day 4, rendering both doses unsuitable for our purposes. Conversely, the 175 mg/kg dose induced diabetes in all the animals, with a 100% survival rate. We then evaluated the change in blood glucose levels before and after administering this dose of STZ. Figure 3A shows that the baseline blood glucose value in female mice before STZ administration was 5.7 ± 0.3 mmol/L. By day three, however, the blood glucose value of the study animals was 11.6 ± 1.1 mmol/L, indicating hyperglycemia or experimental diabetes. This hyperglycemic state persisted and increased to 24.3 ± 2.2 mmol/L by day 6. Intraperitoneal administration of the citrate buffer (STZ vehicle), on the other hand, did not induce significant changes in blood glucose levels in the evaluated animals.

In the NORT, the discrimination index indicates an animal’s ability to distinguish between familiar and unfamiliar objects. This is considered a measure of memory and recognition. The test is based on the assumption that animals with intact memories will spend more time exploring new objects than familiar ones. A reduced or negative discrimination index usually indicates memory impairment. Figure 3B shows that the recognition memory processes of non-diabetic female mice remain intact during the metestrus/diestrus phase. This is evident from the significant increase in the discrimination index during the test phase (day 6) compared to the training phase (day 5). This indicates that cognitive processes are functioning properly. However, two differences were observed in diabetic mice. First, the discrimination indices in the training and test phases were similar. Second, the indices were statistically lower than those of non-diabetic mice in both phases. These results suggest that diabetes impairs recognition memory in female diabetic mice.

### 3.3. Effect of Agmatine on Memory and Locomotor Activity of Diabetic Mice

**Figure 4:**
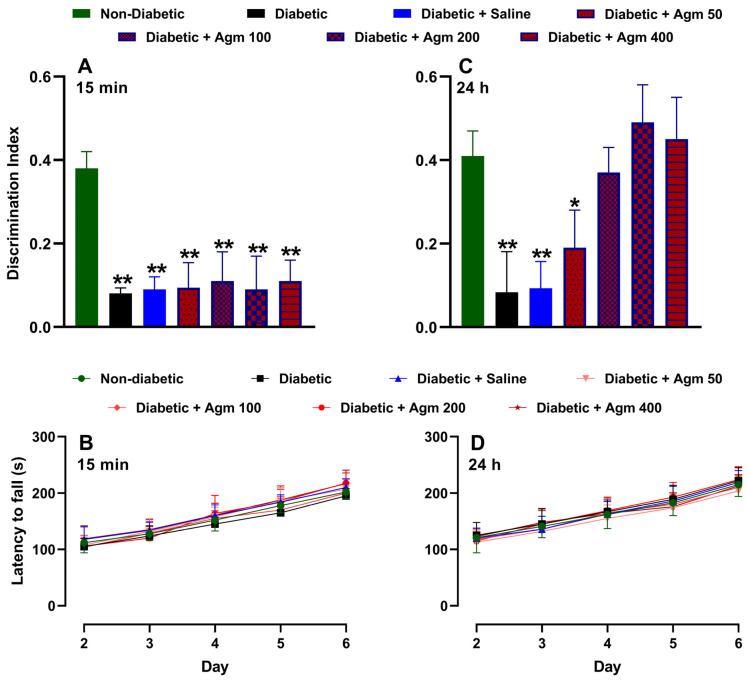
Effect of agmatine (mg/kg) on recognition memory or locomotor activity in diabetic female mice administered intraperitoneally 15 min ((**A**) and (**B**), respectively) and 24 h ((**C**) and (**D**), respectively) before novel object recognition test phase was performed (*n* = 6 for each group). *, *p* < 0.05; **, *p* < 0.01 versus non-diabetic mice.

Figure 4 shows the effects of agmatine on recognition memory and locomotor activity in female diabetic mice. As previously described, Figure 4A illustrates that the discrimination index on day 6 is higher for non-diabetic mice than for diabetic mice. As expected, administering a saline solution does not increase the discrimination index of diabetic female mice. Similarly, administering agmatine (50–200 mg/kg, i.p.) did not significantly alter the discrimination index of diabetic female mice. It is important to note that all treatments were administered 15 min before the NORT testing phase. We also analyzed the locomotor activity of the same animals on days 2–6. On days 4–6, this assay was carried out after the NORT. Figure 4B shows that locomotor activity increased in a time-dependent manner in all evaluated groups. However, there were no significant differences in locomotor activity among non-diabetic, diabetic, and agmatine-treated diabetic female mice. Furthermore, administering agmatine 24 h before the NORT testing phase improved the recognition memory of diabetic female mice in a dose-dependent manner up to 200 mg/kg (Figure 4C). The greatest memory recovery was achieved with a dose of 200 mg/kg, and higher doses did not result in greater cognitive performance. Notably, the locomotor activity with this pharmacological approach was similar to that previously described (Figure 4D).

### 3.4. Effect of Agmatine on Blood and Brain Biochemical Markers in Diabetic Female Mice

**Figure 5:**
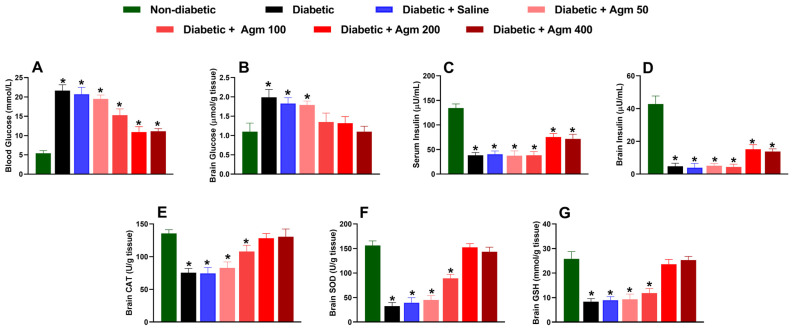
Effect of agmatine (Agm; 50–400 mg/kg, intraperitoneally) on biochemical markers such as (**A**,**B**) blood and brain glucose, (**C**,**D**) serum and brain insulin, (**E**) catalase (CAT), (**F**) superoxide dismutase (SOD), and (**G**) glutathione (GSH) in diabetic female mice (*n* = 6 for each group). *, *p* < 0.05 versus non-diabetic mice.

After conducting behavioral and locomotor studies, we collected blood and brain samples to examine the impact of agmatine on biochemical parameters, such as glucose and insulin levels, in the blood and brain. We also analyzed the effect of agmatine on molecules with cerebral antioxidant capacity, such as CAT, SOD, and GSH, in female diabetic mice. Our results revealed that agmatine significantly reduced blood glucose concentration in diabetic mice at a dose of 100 mg/kg or higher (Figure 5A). The greatest reduction in blood glucose was obtained at 200 and 400 mg/kg. However, it is important to note that agmatine does not fully reverse the increases in blood glucose caused by STZ to levels seen in non-diabetic mice. Regarding brain glucose concentration, we found that agmatine reversed the increase in glucose completely at doses of 100–400 mg/kg (Figure 5B).

STZ administration was found to significantly decrease insulin levels in both the serum and the brain (Figure 5C,D). These effects were not prevented by agmatine administration (50–400 mg/kg). Nevertheless, higher doses of agmatine (200–400 mg/kg) significantly increased insulin levels in blood and brain homogenates compared to those of untreated diabetic animals.

Additionally, agmatine administration increased CAT (Figure 5E), SOD (Figure 5F), and GSH (Figure 5G) levels in the brains of diabetic female mice in a dose-dependent manner. Notably, the decrease in antioxidant capacity in the brains of diabetic mice was completely reversed at 200 and 400 mg/kg. Furthermore, blood and cerebral glucose and insulin levels as well as cerebral CAT, SOD, and GSH levels remained unchanged in diabetic rats treated with saline.

### 3.5. Effect of Several Antagonists on Agmatine-Induced Improvements in Memory and Biochemical Markers in Female Mice

For our study on the mechanisms involved in such responses, we selected a dosage of 200 mg/kg of agmatine because it produced the best results in behavioral and biochemical studies.

**Figure 6:**
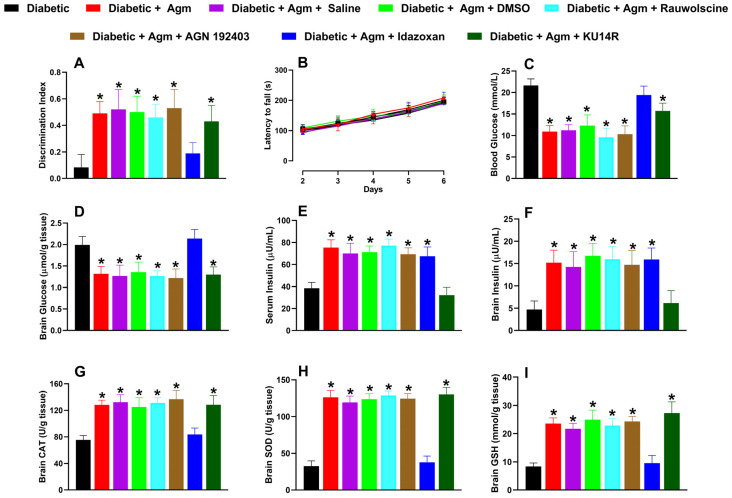
Effect of vehicle controls (saline and dimethyl sulfoxide, DMSO) and specific antagonists, rauwolscine (α_2_; 0.3 mg/kg, i.p.), AGN 192403 (I_1_; 3 mg/kg, i.p.), idazoxan (I_2_; 3 mg/kg, i.p.), and KU14R (I_3_; 8 mg/kg, i.p.), on (**A**) recognition memory, (**B**) locomotor activity, and biochemical markers such as (**C**,**D**) blood and brain glucose, (**E**,**F**) serum and brain insulin, (**G**) catalase [CAT], (**H**) superoxide dismutase [SOD], and (**I**) glutathione [GSH]) in diabetic female mice treated with agmatine (Agm; 200 mg/kg, i.p.) (*n* = 6 for each group). * *p* < 0.05 vs. diabetic mice.

Of the antagonists tested in diabetic animals, only idazoxan (3 mg/kg) reversed the effects induced by agmatine on the following: (1) recognition memory (Figure 6A); (2) blood and brain glucose levels (Figure 6C and 6D, respectively); and (3) increases in catalase (CAT; Figure 6G), superoxide dismutase (SOD; Figure 6H), and glutathione (GSH; Figure 6I). Additionally, only KU14R administration abolished agmatine-induced increases in serum (Figure 6E) and brain tissue (Figure 6F) insulin levels. None of the antagonists affected the locomotor activity of diabetic female mice (Figure 6B).

### 3.6. Correlation Patterns Between Metabolic Parameters, Oxidative Stress, and Cognitive Function

The correlation matrix (Table 2) reveals significant interactions among metabolic parameters, oxidative stress markers, and cognitive function. The discrimination index, an indicator of cognitive ability, shows significant positive correlations with the antioxidant enzymes CAT (r = 0.609), SOD (r = 0.691), and GSH (r = 0.611). These results suggest that maintaining the integrity of the antioxidant system is essential for preserving neural function. Conversely, robust negative correlations are observed between blood glucose and these antioxidants: CAT (r = −0.814), SOD (r = −0.827), and GSH (r = −0.726). This indicates that hyperglycemia induces systemic oxidative stress. This adverse relationship extends to insulin regulation as well, where blood glucose inversely correlates with serum (r = −0.813) and brain (r = −0.755) insulin levels. This reflects peripheral and central insulin resistance.

At the brain level, there is a moderate negative correlation between glucose levels and the discrimination index (r = −0.295). Additionally, strong negative correlations were found between glucose levels and the antioxidants CAT (r = −0.685) and SOD (r = −0.649). These results imply that an accumulation of glucose in neural tissue exacerbates oxidative damage and impairs cognitive function. The strong correlation between serum and brain insulin levels (r = 0.892) highlights the interconnectedness of the peripheral and central compartments in metabolic homeostasis. Antioxidants exhibit extremely strong correlations with each other (CAT/OD: r = 0.956; CAT/GSH: r = 0.929; SOD/GSH: r = 0.943), indicating a cooperative oxidative defense network.

## 4. Discussion

This study provides compelling evidence that agmatine has neuroprotective effects in female diabetic mice. These effects include improved recognition memory, modulation of glucose metabolism, and increased antioxidant capacity in the brain. Our findings are consistent with previous studies on the antioxidant and cognitive-enhancing properties of agmatine in male rodents and models of neurodegenerative diseases [6,12,16]. Notably, our research extends these observations to diabetic female mice during the metestrus/diestrus phases when estrogen levels decrease and the risk of cognitive impairment increases.

As mentioned above, STZ is a nitrosourea that destroys pancreatic cells by alkylating deoxyribonucleic acid (DNA) and depleting nicotinamide adenine dinucleotide/adenosine triphosphate (NAD+/ATP). STZ selectively enters β cells via the GLUT-2 transporter, consequently inducing experimental insulin-dependent (type 1) diabetes [33]. This diabetes mimics many of the complications observed in humans with diabetes. The main cytotoxic effect of STZ-induced diabetes is the potential development of tumors in the kidneys and liver [21]. However, macroscopic observation of these organs showed no evidence of tumor development. Consistent with this finding, locomotor activity was not altered in these animals. These results confirm that STZ induces hyperglycemia, but not tumors, in mice under the reported conditions.

Consistent with previous studies [34,35], our diabetic model revealed that hyperglycemia impairs memory. This decline in memory was accompanied by substantial decreases in cerebral antioxidant defenses (CAT, SOD, and GSH) in the brain, as well as decreased serum and brain insulin levels. Agmatine at a dosage of 200 mg/kg effectively restored recognition memory and reversed key biochemical alterations in diabetic female mice, including elevated glucose levels and reduced antioxidant enzyme activity. These beneficial effects were not accompanied by changes in locomotor activity. In behavioral studies that assess cognitive functions, such as memory, it is crucial to eliminate the possibility of interference from motor impairments. Therefore, a motor coordination test was included. This test is commonly used to detect deficits in coordination, muscle tone, and fine motor function [36]. This ensures that factors affecting performance on tasks such as the NORT are eliminated. It also ensures comparable motor performance between experimental groups. This allows us to attribute any observed differences in exploration or discrimination indices to actual mnemonic processes rather than physical limitations or nonspecific effects of the treatment. In our study, rotarod results showed that neither induced diabetes nor agmatine administration (at any evaluated dose) significantly altered motor coordination or baseline locomotor activity. These results support agmatine’s effectiveness in improving memory.

Notably, we observed a dose-dependent effect up to 200 mg/kg; beyond this dosage, no additional cognitive enhancement occurred, suggesting a possible ceiling effect. Several factors may explain this phenomenon. First, agmatine interacts with multiple receptor systems, including imidazoline receptors, α_2_-adrenoceptors, and NMDA receptors [6]. At supraphysiological concentrations, agmatine may activate nonspecific or opposing pathways that counteract its beneficial effects. For instance, excessive modulation of NMDA receptor activity could impair synaptic plasticity or induce excitotoxicity in certain contexts [37]. Second, high doses of agmatine may saturate its transport or metabolic pathways [38], limiting the concentrations that reach the central nervous system (CNS) beyond a certain threshold. Third, high doses may activate negative feedback mechanisms or receptor desensitization processes that reduce pharmacological efficacy. Similar nonlinear dose–response profiles have been reported for agmatine in other neurobehavioral studies [39,40], supporting the idea that higher doses do not necessarily lead to a greater effect. Therefore, 200 mg/kg appears to be the optimal dose for eliciting cognitive benefits in this model.

Agmatine is a polar compound with a limited ability to cross the blood–brain barrier (BBB). However, pharmacokinetic and molecular imaging studies have identified mechanisms that facilitate its transport to the CNS. Evidence suggests that agmatine enters the CNS via an active, saturable, pH-dependent process through polyamine transporters, specifically organic cation transporter 2 (OCT2) and the extraneuronal monoamine transporter (EMT) [38]. In vivo studies in rodents confirm its brain biodistribution [41,42]. However, the optimal dose used here is higher than those commonly reported. Agmatine is typically administered at a dose of 10–100 mg/kg to male or non-diabetic rodents either acutely or subchronically [6,13,43]. This difference may be explained by sex- and disease-specific factors, including greater oxidative stress, insulin deficiency, and increased susceptibility to neurodegeneration due to reduced estrogen levels. It is important to note that a 200 mg/kg dose of agmatine remains within the nontoxic range [44,45].

The timing of agmatine administration (15 min and 24 h before the NORT) was based on its pharmacokinetic profile [42] and experimental criteria that aimed at distinguishing between immediate effects and sustained effects, or those mediated by slower mechanisms. Intraperitoneal administration of agmatine at a dose of 200 mg/kg produced significant improvements in recognition memory in diabetic female mice when given 24 h prior to testing but not when administered 15 min before the task. This time-dependent effect suggests that although agmatine rapidly distributes to the brain (t_max_ = 15 min) [42], this brief timeframe is insufficient to trigger its neuroprotective or memory-enhancing actions. Once in the brain, however, agmatine has a longer half-life of approximately 8.5 h [42]. This enables agmatine to initiate intracellular signaling cascades and gene expression changes that require time to manifest. Consequently, a single 200 mg/kg dose produces effects that extend beyond its immediate presence in the bloodstream. These effects ultimately lead to improved recognition memory, which is observed 24 h after administration. These processes are not immediate and may require hours to alter the necessary gene expression, protein synthesis, and enzymatic activity necessary for neuroprotection and memory consolidation. In contrast, the absence of cognitive benefits 15 min after administration implies that agmatine does not act solely through rapid neurotransmitter modulation. Furthermore, the i.p. route ensures systemic distribution, including access to the central nervous system, without the complications associated with oral bioavailability or gastrointestinal tract degradation [42,46,47]. Future studies should compare alternative administration routes, such as intracerebroventricular, oral, or subcutaneous administration to better define the most effective and clinically viable method of delivery for achieving cognitive benefits.

The effects of agmatine on glucose metabolism appear to be mediated by several converging mechanisms. First, agmatine activates imidazoline receptors, particularly I_2_, in the adrenal gland [48,49,50] to lower blood glucose via β-endorphin/opioid µ-receptors/glucose transporter 4 (GLUT-4) and enhance glucose transporter 2 (GLUT-2) in the liver [51]. Second, I_3_ receptor activation in pancreatic β-cells enhances insulin release [31], which can account for the observed increase in both serum and brain insulin levels following agmatine administration. This facilitates glucose clearance from the bloodstream. Additionally, agmatine activates I_2_ receptors in the central nervous system, which may improve insulin signaling and glucose utilization at the neuronal level [52], thereby reducing brain glucose concentration.

Agmatine is known for its antioxidant properties. It increases the expression and activity of important antioxidant enzymes, such as CAT and SOD, as well as the production of the antioxidant peptide glutathione (GSH). This occurs through the activation of signaling pathways such as Nrf2 (nuclear factor erythroid 2-related factor 2) [10,53,54]. Nrf2 is a transcription factor that regulates the expression of various cytoprotective and antioxidant genes. Agmatine promotes Nrf2 activity, thereby enhancing the cellular defense system against reactive oxygen species (ROS), which are elevated in diabetic conditions. Agmatine also inhibits nitric oxide synthase (NOS) [55] and modulates polyamine metabolism [56,57,58], leading to decreased formation of advanced glycation end-products and reduced oxidative damage. These factors are commonly elevated in diabetes. Restoring antioxidant enzyme levels protects neural tissue from oxidative damage and improves cognitive performance.

Although the selection of antagonists in this study was pharmacologically sound, it is important to note that some of these compounds may not act exclusively as pure antagonists. Several reports have indicated that drugs such as idazoxan, rauwolscine, and KU14R may display partial agonist activity or interact with other receptor systems. This could potentially complicate data interpretation. For example, although idazoxan is commonly used as an imidazoline I_2_ receptor antagonist, it has also been shown to exhibit partial agonist properties at α_2_-adrenoceptors and moderate affinity for I_1_ receptors, depending on the dose and tissue [59]. Similarly, rauwolscine, traditionally classified as a selective α_2_-adrenoceptor antagonist, may also bind to serotonin (5-HT_1A_ and 5-HT_2B_) receptors [60,61]. AGN 192403 and KU14R, which were used to block I_2_ and I_3_ receptors in this study, have been reported to act as a partial agonist of their respective receptors [62,63].

Based on the above, idazoxan (an I_2_ receptor antagonist) was shown to abolish agmatine-induced improvements in memory and antioxidant enzyme activity, as well as reductions in blood and brain glucose levels. These findings support the hypothesis that I_2_ receptor activation is essential for agmatine’s therapeutic effects in this model. This hypothesis aligns with recent evidence linking imidazoline receptors to neuroprotection and metabolic regulation [64,65,66]. On the other hand, agmatine is a pleiotropic molecule that interacts with α_2_-adrenoceptors, imidazoline, and NMDA receptors, as well as other molecular targets. Thus, the observed cognitive and biochemical improvements likely result from the convergence of multiple receptor-mediated pathways rather than from an I_2_ receptor mechanism. The unknown molecular nature of imidazoline receptors hinders the development of selective agonists. Currently, there is no selective I_2_ agonist that can be used as a positive control. This limits the mechanistic conclusions that can be drawn about the specific role of this receptor. However, several research groups have undertaken the task of synthesizing more selective ligands based on functional studies [30,65,67]. In the near future, using these types of ligands will be essential for discerning the actual contribution of each imidazoline receptor.

Notably, KU14R prevented agmatine from restoring insulin levels, suggesting that I_3_ receptors modulate insulin signaling. In contrast, blocking either the α_2_-adrenoceptor or the I_1_ receptor blockade did not significantly affect the observed outcomes. These results imply that these receptors are not critical for agmatine’s cognitive or metabolic effects in diabetic female mice. However, secondary interactions may still influence the overall pharmacological profile, especially under diabetic or hormonally altered conditions. Future studies using mouse knockout models or more selective ligands may refine our understanding of these receptor-mediated mechanisms. Additionally, further research is needed to investigate the long-term effects of agmatine on type 1 and type 2 models of diabetes and determine if its benefits are sustained over time. Studies on ovariectomized or estrogen-supplemented models could also clarify the role of sex hormones in agmatine’s efficacy. Elucidating the downstream signaling pathways activated by imidazoline I_2_ and I_3_ receptors in the brain could also provide new targets for pharmacological intervention.

**Figure 7:**
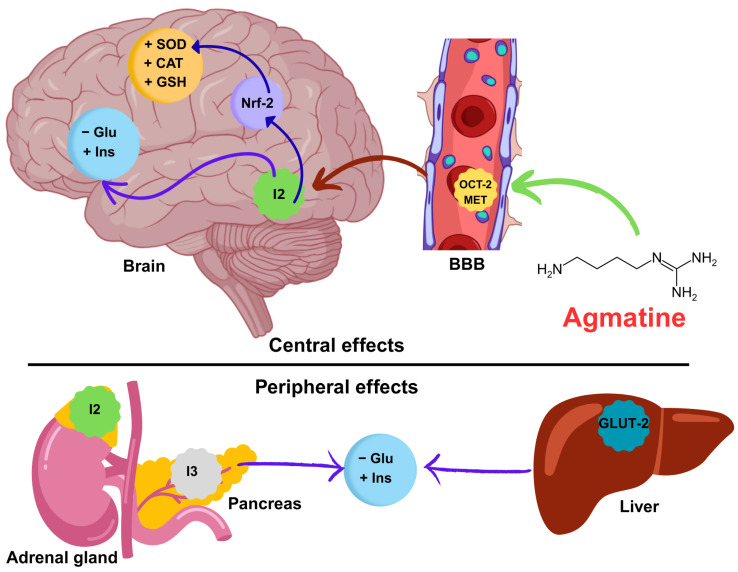
Mechanistic hypothesis of the neuroprotective effect of agmatine on memory in diabetic mice. Agmatine crosses the blood–brain barrier (BBB) via the organic cation transporter (OCT-2) and the extraneuronal monoamine transporter (EMT). Once in the brain, agmatine activates the transcription factor Nrf-2, increasing the synthesis of antioxidant enzymes (SOD, CAT, and GSH). Agmatine also improves glucose (Glu) and insulin (Ins) homeostasis. Simultaneously, agmatine exerts peripheral effects on the adrenal gland and pancreas and liver (modulation of GLUT-2), attenuating systemic hyperglycemia. These central and peripheral synergistic mechanisms counteract oxidative stress and metabolic dysfunction, thereby restoring cognitive function. Abbreviations: SOD (superoxide dismutase), CAT (catalase), GSH (glutathione), Glu (glucose), Ins (insulin), OCT-2 (organic cation transporter 2), GLUT-2 (glucose transporter 2).

Correlation analysis revealed a positive association between cognitive performance, as measured by the discrimination index, and antioxidant enzymes (CAT, SOD, and GSH). These results support the idea that maintaining an oxidative balance is important for preserving neural function in people with diabetes. However, strong negative correlations between blood glucose levels and antioxidant and insulin levels suggest that hyperglycemia promotes oxidative stress and insulin resistance in both the periphery and the central nervous system. These results are consistent with previous studies showing that impaired insulin signaling and increased oxidative damage lead to cognitive impairment in diabetes [68]. The high intercorrelation among antioxidant markers indicates the presence of a coordinated redox defense network that may be strengthened by agmatine’s established antioxidant properties. Overall, the data support the hypothesis that agmatine’s cognitive benefits are mediated by improved oxidative status and glucose–insulin homeostasis. Taking all the evidence into account, we propose a mechanistic pathway integrating our findings with the existing literature. This pathway emphasizes the correlation between increased antioxidant enzyme activity, improved insulin signaling, and reduced hyperglycemia after agmatine treatment. The pathway involves central and peripheral sites of action (see Figure 7).

Despite the promising results observed in this study, several limitations must be noted. First, the acute treatment model limits our understanding of the long-term effects and safety profile of agmatine, particularly in the context of chronic diabetic neurodegeneration. Second, intraperitoneal administration does not reflect a typical clinical scenario, as oral delivery would be more feasible. This raises questions about bioavailability and effective dosing in humans. From a translational perspective, however, the observed cognitive and biochemical improvements suggest therapeutic potential. Further studies are needed to evaluate pharmacokinetics, optimal dosing strategies, and potential interactions with standard antidiabetic treatments in humans. Addressing these aspects in future research is essential to bridge the gap between preclinical findings and clinical application.

## 5. Conclusions

This study demonstrates that agmatine improves recognition memory and antioxidant balance in female diabetic mice during the low-estrogen phase of their estrous cycle. These effects were associated with increased insulin levels, reduced blood and brain glucose concentrations, and enhanced antioxidant enzyme activity in the brain such as superoxide dismutase, catalase, and glutathione. Pharmacological blockade experiments suggest that imidazoline I_2_ and I_3_ receptors play a role in mediating these outcomes. However, given the pharmacological promiscuity of agmatine and the non-selective nature of the antagonists used, the mechanistic interpretation of these findings should remain tentative until further studies employing more selective tools or genetic models are conducted. Nevertheless, these mechanisms may be essential for protecting the neural circuits responsible for memory and cognition, particularly in cases of diabetes-induced neurodegeneration. Notably, agmatine does not alter the locomotor activity in diabetic mice, suggesting direct neuroprotective action.

## Figures and Tables

**Table 1:** Stages of the estrous cycle of the female mice in each subgroup (*n* = 6) of the experimental protocol at the time of the novel object recognition test phase (day 6). Each subgroup consists of six animals. STZ, streptozotocin; CB, citrate buffer; ND, non-diabetic; D, diabetic; Agm, agmatine; DMSO, dimethyl sulfoxide. The percentage (%) is based on six mice per group.

Treatment	Proestrus	Estrus	Metestrus	Diestrus
	%			
Group 1				
STZ	0	0	16.7	83.3
CB	0	0	33.3	66.7
Group 2				
ND	0	0	0	100
D	0	0	33.3	66.7
D-Saline	0	0	16.7	83.3
D-Agm 50	0	0	33.3	66.7
D-Agm 100	0	0	16.7	83.3
D-Agm 200	0	0	0	100
D-Agm 400	0	0	16.7	83.3
Group 3				
ND-Saline	0	0	33.3	66.7
D	0	0	0	100
D-Saline	0	0	16.7	83.3
D-Agm 50	0	0	33.3	66.7
D-Agm 100	0	0	0	100
D-Agm 200	0	0	16.7	83.3
D-Agm 400	0	0	33.3	66.7
Group 4				
D-Agm 200 + Saline	0	0	16.7	83.3
D-Agm 200 + DMSO	0	0	0	100
D-Agm 200 + rauwolscine	0	0	33.3	66.7
D-Agm 200 + AGN 192403	0	0	16.7	83.3
D-Agm 200 + Idazoxan	0	0	0	100
D-Agm 200 + KU14R	0	0	0	100

**Table 2:** Pearson correlation matrix (r) between cognitive, metabolic, and oxidative stress parameters. The discrimination index reflects the cognitive ability of diabetic mice. Catalase (CAT), superoxide dismutase (SOD), and glutathione (GSH) are markers of the antioxidant defense system.

Pearson Correlation (r)								
Variables	Discrimination Index	Blood Glucose	Brain Glucose	Serum Insulin	Brain Insulin	CAT	SOD	GSH
Discrimination index	1.000							
Blood glucose	−0.552	1.000						
Brain glucose	−0.295	0.803	1.000					
Serum insulin	0.301	−0.813	−0.468	1.000				
Brain insulin	0.228	−0.755	−0.514	0.892	1.000			
CAT	0.609	−0.814	−0.685	0.696	0.636	1.000		
SOD	0.691	−0.827	−0.649	0.711	0.641	0.956	1.000	
GSH	0.611	−0.726	−0.567	0.644	0.612	0.929	0.943	1.000

## Data Availability

The original data presented in the study are openly available in Open Science Framework (OSF): “https://osf.io/kzh38/files/osfstorage (accesed on 24 June 2025)”.

## Supplementary Materials

The following supporting information can be downloaded at: https://www.mdpi.com/article/10.3390/antiox14070837/s1, Table S1. Checklist of the ARRIVE Guidelines, Version 2.0.
